# PtrWRKY73, a salicylic acid-inducible poplar WRKY transcription factor, is involved in disease resistance in *Arabidopsis thaliana*

**DOI:** 10.1007/s00299-015-1745-5

**Published:** 2015-01-28

**Authors:** Yanjiao Duan, Yuanzhong Jiang, Shenglong Ye, Abdul Karim, Zhengyi Ling, Yunqiu He, Siqi Yang, Keming Luo

**Affiliations:** 1 Key Laboratory of Eco-environments of Three Gorges Reservoir Region, Ministry of Education, Chongqing Key Laboratory of Transgenic Plant and Safety Control, School of Life Sciences, Institute of Resources Botany, Southwest University, No. 1, Tiansheng Road, Beibei, 400715 Chongqing China; 2 Key Laboratory of Adaptation and Evolution of Plateau Biota, Northwest Institute of Plateau Biology, Chinese Academy of Sciences, Xining, 810008 China

**Keywords:** *Populus*, WRKY, Transcription factor, Pathogen, SA

## Abstract

*****Key message***:**

**A salicylic acid-inducible WRKY gene,** ***PtrWRKY73,*** **from** ***Populus trichocarpa*** **, was isolated and characterized. Overexpression of** ***PtrWRKY73*** **in** ***Arabidopsis thaliana*** **increased resistance to biotrophic pathogens but reduced resistance against necrotrophic pathogens.**

**Abstract:**

WRKY transcription factors are commonly involved in plant defense responses. However, limited information is available about the roles of the WRKY genes in poplar defense. In this study, we isolated a salicylic acid (SA)-inducible WRKY gene, *PtrWRKY73,* from *Populus trichocarpa*, belonging to group I family and containing two WRKY domains, a D domain and an SP cluster. *PtrWRKY73* was expressed predominantly in roots, old leaves, sprouts and stems, especially in phloem and its expression was induced in response to treatment with exogenous SA. PtrWRKY73 was localized to the nucleus of plant cells and exhibited transcriptional activation. Overexpression of *PtrWRKY73* in *Arabidopsis thaliana* resulted in increased resistance to a virulent strain of the bacterial pathogen *Pseudomonas syringae* (*Pst*DC3000), but more sensitivity to the necrotrophic fungal pathogen *Botrytis cinerea*. The SA-mediated defense-associated genes, such as *PR1*, *PR2* and *PAD4*, were markedly up-regulated in transgenic plants overexpressing *PtrWRKY73*. *Arabidopsis* non-expressor of *PR1* (*NPR1*) was not affected, whereas a defense-related gene *PAL4* had reduced in *PtrWRKY73* overexpressor plants. Together, these results indicated that PtrWRKY73 plays a positive role in plant resistance to biotrophic pathogens but a negative effect on resistance against necrotrophic pathogens.

**Electronic supplementary material:**

The online version of this article (doi:10.1007/s00299-015-1745-5) contains supplementary material, which is available to authorized users.

## Introduction

Plants are constantly challenged by a variety of microbial pathogens like fungi and bacteria. Complex defense mechanisms have evolved in plants to protect themselves against the attack of pathogens. Upon pathogen infection, plants rapidly activate defense responses mediated by multiple signal transduction pathways (Durrant and Dong 2004; Jones and Dangl 2006). Phytohormones are important components of different signaling pathways involved in plant defense (Rivas-San Vicent and Plasencia 2011). In many cases, pathogen-induced accumulation of salicylic acid (SA) leads to a rapid increase in the levels of reactive oxygen species (ROS) (Chen et al. 1993; Baker and Orlandi 1995; Lamb and Dixon 1997; Klessig et al. 2000). The change in the cellular redox potential resulted in the translocation of NONEXPRESSOR OF PR1 (NPR1) into the nucleus and enhanced transcriptional co-activator level, such as TGA transcription factors which activated SA-responsive pathogenesis-related genes (*PRs*) to trigger systemic acquired resistance (SAR) against biotrophic pathogen (Uknes et al. 1992; Sticher et al. 1997; Dong 2004; Zheng et al. 2006; Loake and Grant 2007). In other cases, necrotrophic pathogens such as *Alternaria brassicicola* or *Botrytis cinerea* can induce defense responses characterized by jasmonic acid (JA)- and ethylene (ET)-dependent signal transduction pathways (Thomma et al. 1999). It has been well demonstrated that these signaling pathways are not separate cascades, but can interact with each other (Reymond and Farmer 1998; Kunkel and Brook 2002). For example, the JA-signaling mutants, *mpk4* and *coi1*, show enhanced SA accumulation and signaling (Petersen et al. 2000; Kloek et al. 2001), whereas blocking SA accumulation in pathogen-infected plants can improve JA signaling (Spoel et al. 2003).

Plant defense responses are involved in defense-response gene activation upon pathogen infection (Rushton and Somssich 1998). In SA-mediated defense signaling pathway, expression of certain *PR* genes are induced to increase resistance to biotrophic and hemibiotrophic pathogens (Kunkel and Brook 2002). *NPR1*, whose nuclear localization is required for SA signaling, mediates pathogen resistance in many plants (Spoel et al. 2003). The expression of the JA-responsive genes *LOX2*, *PDF1.2*, and *VSP* was up-regulated in response to infection by *Pseudomonas* *syringae* pv. tomato DC3000 (*Pst*DC3000) in the plants unable to accumulate SA (Penninckx et al. 1998). In *Arabidopsis*, *PDF1.2* is used as a marker for JA defense signaling pathways and is often correlated with resistance to necrotrophic pathogens (Thomma et al. 1998). In *planta*, products of pathogen-induced genes include key enzymes for phytoalexin biosynthesis (Ferrari et al. 2007; Ren et al. 2008) and those encoding transcription regulatory factors involved in signal perception and transduction of plant defense responses. Between them, transcriptional regulation of plant genes is a central step in plant defense responses. Therefore, elucidation of the complex regulatory mechanisms for controlling defense gene expression among plant species is important to understanding the molecular basis of plant–pathogen interactions.

WRKY transcription factors have been extensively studied with regard to their roles in regulation of genes associated with plant defense responses (Ulker and Somssich 2004; Eulgem and Somssich 2007). This gene family had a lineage-specific expansion during the course of plant evolution, and consists of 45 members in barley (Mangelsen et al. 2008), 74 in *Arabidopsis* (Ulker and Somssich 2004), 81 in rice (Xie et al. 2005) and at least 100 in *Populus trichocarpa* (He et al. 2012; Jiang et al. 2014). All products of *WRKY* genes contained either one or two WRKY domains, which comprised of a highly conserved amino acid sequence, WRKYGQK, following a zinc-finger motif CX_4–7_-CX_23–28_-HX_1–2_-(H/C) (Eulgem et al. 2000). Further, phylogenetic analysis of WRKY domains suggests that they evolved from an ancestral group IIc-like WRKY early in the eukaryote lineage (Brand et al. 2013). The conservation of the WRKY domain interacted with a remarkable conservation of the *cis*-acting W-box [(T)(T)TGAC(C/T)] containing the invariant TGAC core, which is required for function and WRKY binding (Eulgem et al. 2000).

Increasing evidence demonstrates that WRKY proteins participate widely in plant immunity. In *Arabidopsis*, 49 of the 74 AtWRKY genes were modulated in plants infected by *P. syringae* or treated by SA (Dong et al. 2003). W-boxes distribute in the promoters of target genes correlated with plant defense mediated by SA and JA signaling pathway, such as *NPR1*, *PR2*, *PR10*, *VSP1* and *VSP2* (Despres et al. 1995; Rushton et al. 1996; Yu et al. 2001; Li et al. 2004). Interestingly, W-boxes were also found in the promoters of some WRKY genes. For example, a cluster of W-boxes in the promoter of *AtWRKY18* acted as negative regulatory elements for its inducible expression (Chen and Chen 2002). Furthermore, some WRKY genes regulate the expression of genes involved in the biosynthesis of defense-related phytohormones. WRKY28 and WRKY46 are transcriptional activators of *ISOCHORISMATE SYNTHASE 1* (*ICS1*) and *AVR*_*PPHB*_ *SUSCEPTIBLE 3* (*PBS3*), which are associated with SA synthesis and SA-glucoside accumulation in *Arabidopsis*, respectively (van Verk et al. 2011). On the contrary, AtWRKY70 and AtWRKY54 play roles as negative regulators of SA biosynthesis (Wang et al. 2006). WRKY proteins also regulate antagonistic relationships between different defense pathways. For instance, mutation of *AtWRKY33* resulted in enhanced susceptibility to the necrotrophic fungal pathogens, *Botrytis cinerea* and *Alternaria brassicicola*. Ectopic overexpression of *AtWRKY33* not only increased resistance to necrotrophic fungi, but also made plants more susceptible to biotrophic pathogen *P. syringae* (Zheng et al. 2006). AtWRKY70 also functions as a repressor of JA-responsive genes and an activator of SA-induced genes, integrating signals from the two antagonistic pathways to modulate resistance to different races of pathogens (Li et al. 2004). In addition, strawberry FaWRKY1 is associated with a strong oxidative burst and glutathione-S-transferase (GST) induction to increase resistance to avirulent strains of *P. syringae* (Encinas-Villarejo et al. 2009). In *Populus*, PtWRKY23 affected resistance to *Melampsora* infection possibly by deregulation of genes that disrupt redox homeostasis and cell wall metabolism (Levée et al. 2009). In a previous study, we found several defense-related *cis*-elements distributed in the promoters of poplar WRKY genes and about 60 WRKY genes were induced or down-regulated by treatments of SA, MeJA and *Marssonina brunnea* (Jiang et al. 2014). However, the functional characterization of these WRKY genes in *Populus*, especially their roles in plant immune responses, is limited.

In this study, we isolated and characterized an SA-inducible gene *PtrWRKY73* from *P. trichocarpa*. Amino acid sequence alignment and phylogenetic analyses showed that PtrWRKY73 is a group I member of the WRKY gene family and is similar to tobacco NtWRKY1 and AtWRKY33. Gene expression profiling in wild-type plants showed that transcripts of *PtrWRKY73* accumulated in roots, old leaves, sprouts and stems (especially phloem). Overexpression of *PtrWRKY73* in *Arabidopsis* resulted in increased resistance to *P. syringae* but more susceptibility to *B. cinerea*. In transgenic lines, overexpressing *PtrWRKY73*, the genes involved in SA signaling pathway, such as *PR1*, *PR2*, *WRKY70*, *PAD4* and C*PR5* were markedly up-regulated, whereas expression of the phenylalanine ammonia-lyase gene *PAL4*, an SA biosynthesis-related gene was decreased. These results indicate that PtrWRKY73 might regulate the SA-mediated defense pathway to mediate resistance to different pathogens in *Arabidopsis*.

## Methods and materials

### Plant material and treatments

*Populus trichocarpa* Torr. and A. Gray and *P. tomentosa* Carr. (clone 741) were grown in a greenhouse at 25 °C under a 14 h light and 10 h dark cycle with supplemental light (4,500 lux). 2-month-old poplars were employed for gene expression analyses. Seeds of *Arabidopsis thaliana* (Columbia 0 ecotype) were kept at 4 °C for at least 2 days before placement in a growth environment to facilitate uniform germination. Seedlings were transferred to pots after 2 weeks of germination on MS plates (Murashige and Skoog 1962). Plants were grown on a 1:1 mixture of vermiculite and peat in an illumination incubator at 22 °C, 80 % relative humidity and a 16 h photoperiod with supplemental light.

SA (5 mM in water) was applied to whole plant of poplar and *Arabidopsis*, respectively. The treated plants were immediately covered with a transparent lid and the leaves were collected after treatments (Li et al. 2004).

*Botrytis cinerea* were cultured, incubated and plant inoculation was performed as described previously (Zheng et al. 2006). Semi-quantitative RT-PCR was performed to quantify the *ACTIN* of *Botrytis* using the specific primers (F: 5′-CGCCCCTGCATTCTACGTCTC-3′; R: 5′-CAAGCTGGAGGATTGACTGGC-3′).

Pathogen inoculations were performed by infiltration of leaves of at least six plants for each treatment with the *Pseudomonas syringae* pv. tomato DC3000 strain (OD_600_ = 0.001 in 10 mM MgCl_2_). Inoculated leaves were harvested 3 d after infiltration and homogenized in 10 mM MgCl_2_. Diluted leaf extracts were plated on King’s B medium supplemented with rifampicin (100 μg/mL), incubated at 25 °C for 2 days before counting the colony-forming units.

### Cloning of *PtrWRKY73*, vector construction and transformation of *Arabidopsis*

The cDNA fragment encoding *PtrWRKY73* was PCR amplified with gene-specific primers (*PtrWRKY73*-F: 5′-ATGGCTGCTTCTTCAGGGAGC-3′; *PtrWRKY73*-R: 5′-CCAAGAACTCCTACGTGCTACG-3′) based on the transcript sequence (Potri.013G153400.1) from the *P. trichocarpa* genome. The PCR was performed using pfu DNA polymerase (Takara, Dalian, China) in a total volume of 50 μL. Cycling conditions were 94 °C for 3 min; 32 cycles of 94 °C for 45 s, 60 °C for 1 min and 72 °C for 2 min, followed by a final extension of 72 °C for 10 min. The PCR products were cloned into the plant binary vector pCXSN (Chen et al. 2009). The resulting vector *p35S:PtrWRKY73*, with the *PtrWRKY73* open reading frame was driven by the cauliflower mosaic virus (CaMV) 35S promoter and the hygromycin phosphotransferase (*Hpt*) gene conferring resistance to hygromycin. The genomic DNA fragment containing an approximate 1.5 kb upstream sequence of *PtrWRKY73* was amplified by the primers: Pro-PtrWRKY73 (F: 5′-GGATCAGTCAAAGAACAAGCTG-3′; R: 5′-GAAGAGGTCATGAAAGGGTAG-3′). The Pro-PtrWRKY73:GUS construct in the binary vector pCXGUS-P (Chen et al. 2009) was made by ligating the PCR-amplified genomic DNA fragments. Finally, these two constructs were transferred into *Agrobacterium tumefaciens* strains EHA105 by the freeze–thaw method. Transformation of *A. thaliana* plants was carried out by the floral dip method (Clough and Bent 1998). Transformants were selected on MS plates supplemented with 30 mg/mL of hygromycin and 50 mg/mL of carbenicillin.

### Sequence comparisons and phylogenetic analysis

Amino acid sequence alignments were performed with DNAMAN software. The phylogenetic tree was constructed by the neighbor-joining method using MEGA 4.1 software (LynnonBiosoft, Quebec, Canada). The accession numbers of the WRKY proteins were kept in Supplementary Table 1.

### Molecular analysis of transgenic plants

Genomic DNA was extracted from 300 mg of both untransformed control and transgenic plants using a CTAB method (Jia et al. 2010). Each PCR mixture (10 μL) contained 5.5 μL GoTaq^R^Green Master Mix (Promega, Beijing, China), 0.25 μL of each primer, 0.5 μL cDNA and 3.5 μL nuclease-free water. PCR analysis was carried out with gene-specific primers that were positioned on the encoding sequence of PtrWRKY73 and the NOS terminal of pCXSN vector. PCR products were resolved on a 1 % (w/v) agarose gel and visualized after ethidium bromide staining under UV light.

### RNA extraction and quantitative real-time PCR and semi-quantitative RT-PCR

Total RNA from fresh tissues of plants was extracted using RNA RNeasy Plant Mini Kit (Qiagen, Germany) according to the manufacturer’s instructions with a modification as reported previously (Jia et al. 2010). Extraction of total RNA from the xylem and phloem tissues was performed as Tian et al. (2013). Samples from at least three plants were pooled for analysis. The total RNA before the cDNA synthesis was treated with RNase-free DNase (TaKaRA, Dalian, China), following the manufacturer’s instructions to avoid any genomic DNA contamination. First-strand cDNA was synthesized from 2 µg RNA with RT-AMV transcriptase (TaKaRa, Dalian, China) in a total volume of 20 µL using oligo (dT)18 at 42 °C for 30 min. *18S* rRNA was used as an internal control. The amplification products of RT-PCR were resolved by 1 % (w/v) agarose gel electrophoresis and visualized with ethidium bromide under UV light to test the expression level of *PtrWRKY73* under various treatments and defense-related genes in transgenic plants. Quantitative real-time PCR and data analysis were performed in a 25 μL reaction volume containing 12.5 μL of SYBR Premix ExTaq^TM^ (TaKaRa, Dalian, China). Differences of genes expression, expressed as fold change relative to control, were calculated using the [Δ][Δ]C_t_ = 2^[Δ]Ct,18S − [Δ]Ct^ method. The gene-specific primers used for semi-quantitative RT-PCR and quantitative real-time PCR analysis were shown in Supplementary Table 2. Real-time PCR analysis was based on at least two biological replicates of each sample and three technical replicates of each biological replicate.

### Subcellular localization

The PCR-amplified cDNA of *PtrWRKY73* using primers of *PtrWRKY73*-pCX-DG-F and *PtrWRKY73*-R were ligated into pCX-DG (Chen et al. 2009) to generate the *PtrWRKY73*-*GFP* construct. The resulting vector was induced into onion epidermal cells by Gene Gun (GJ-1000, SCIENTZ, China). The onion skin was stained with DAPI, and photographed under a light microscope (Olympus BX53).

### Transactivation assay

The ORF of *PtrWRKY73* was amplified by PCR with gene-specific primers and cloned into *Nco*RI/*Bam*HI-digested pGBKT7 vector. The resulting vector, positive control pGBKT7-PtrWRKY73 and negative control pGBKT7 (empty vector) were transformed into yeast strain *Saccharomyces cerevisiae* Gold2. Transformants were grown on synthetic dropout medium (SD medium) lacking tryptophan (Trp) for positive clone selection and then on SD medium lacking tryptophan (Trp), histidine (His) and adenine (Ade) for the transactivation assay, according to the method described previously (Tian et al. 2013).

### GUS staining

GUS staining of T3 lines was performed by immersing seedlings in a staining solution (0.1 M sodium phosphate buffer, pH 7, 2 mM K_4_Fe(CN)_6_, 2 mM K_3_Fe(CN)_6_, 0.2 % Triton X-100, 10 m MEDTA, 2 mM X-Gluc) in 50 mL Falcon tubes for 4 h at 37 °C in the dark and washed with 70 % ethanol to remove chlorophyll (Weigel and Glazebrook 2002).

### Statistical analysis

The Student’s *t* test program (http://www.graphpad.com/quickcalcs/ttest1.cfm) was used for statistical analysis of the data in the experiments of quantitative real-time PCR. In all these experiments, it was found that the quantitative differences between the two groups of data for comparison were statistically significant (*P* < 0.001).

## Results

### Expression of *PtrWRKY73* is induced rapidly by various stresses

**Fig. 1:**
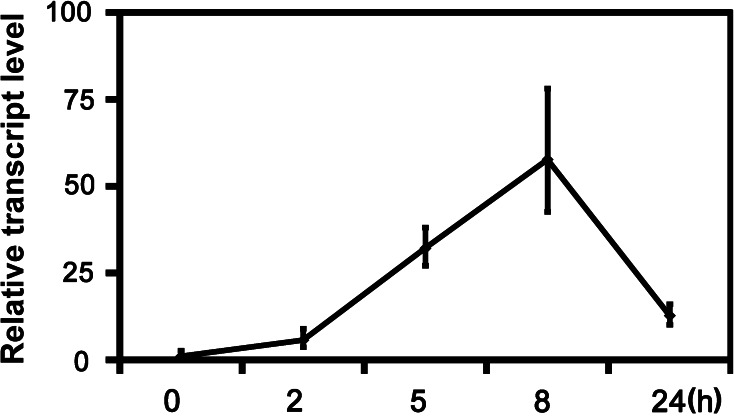
Response of *PtrWRKY73* to exogenous salicylic acid treatments in *Populus trichocarpa* over time. Expression values are relative to time point 0 h. *Error bars* represent three biological replicates

In a previous study, the functions of all poplar WRKY transcription factor family members have been characterized in plant defense and response to various biotic and abiotic stresses (Jiang et al. 2014). A putative WRKY gene, named *PtrWRKY73*, was identified in the *P. trichocarpa* genome, which was significantly induced by the fungus *M. brunnea* f. sp. *multigermtubi* and SA treatment. To confirm whether *PtrWRKY73* was involved in biotic and abiotic stresses in *P. trichocarpa*, expression patterns of *PtrWRKY73* were determine by quantitative real-time PCR over a time course of 24 h after treatment with SA. The *PtrWRKY73* transcript level was significantly increased at 5 h and with a peak at 8 h (Fig. 1). These results indicated that the expression of *PtrWRKY73* could be induced by defense signals and may play an important role in plant resistance to pathogens.

### Isolation and characterization of *PtrWRKY73*

**Fig. 2:**
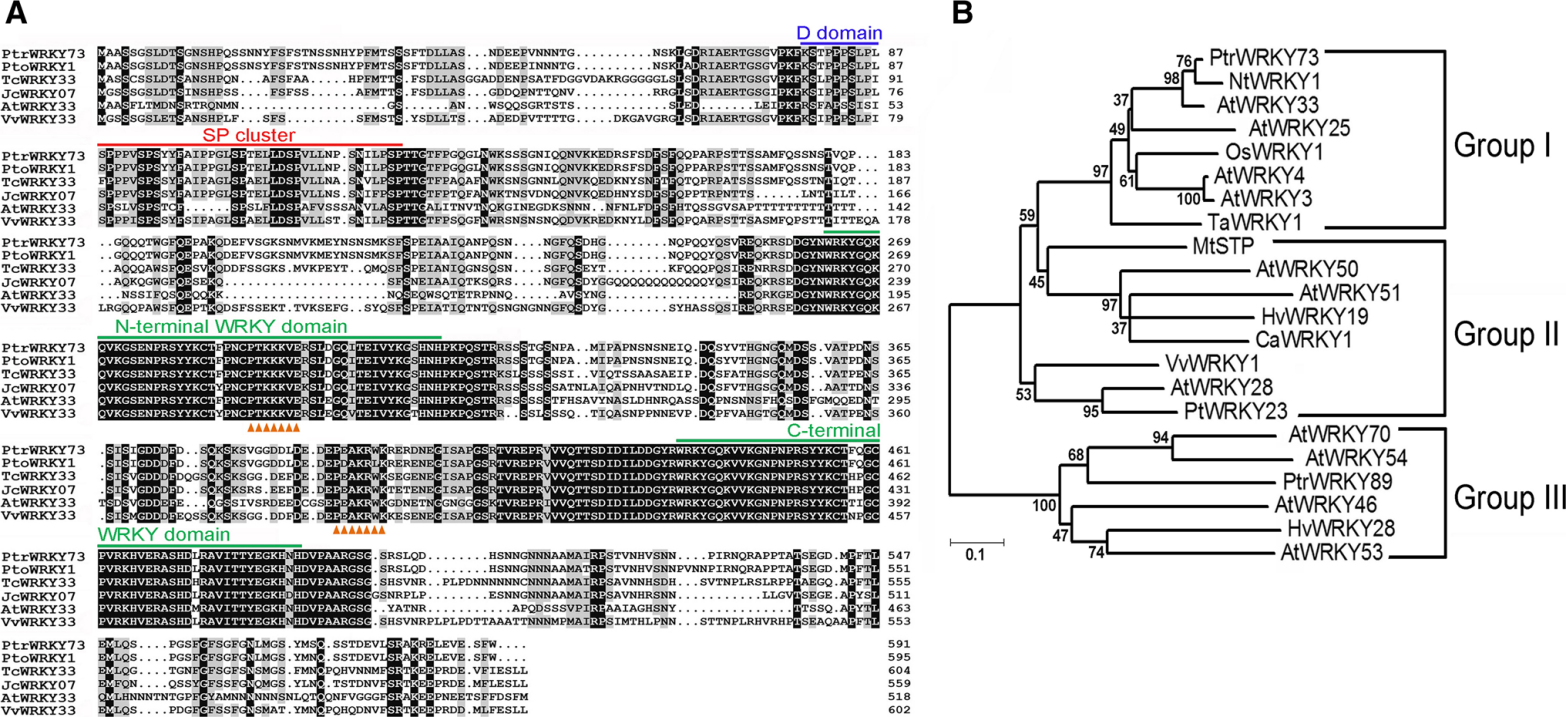
Alignment and phylogenetic analysis of PtrWRKY73 with other WRKY amino acid sequences. **a** Sequence alignment PtrWRKY73 and the other WRKYs. Sequences were from [(*P. tomentosa* × *P. bolleana*) × *P. tomentosa*] (PtoWRKY1), *Theobroma cacao* (TcWRKY33), *J. curcas* (JcWRKY07), *V. vinifera* (VvWRKY33) and *A. thaliana* (ATWRKY33). Identical amino acids were indicated by *white letters* *on a* *black* *background* and conservative amino acids by *black* *on* *a dark gray background*. The N-terminal and C-terminal WRKY domains were highlighted by* green lines*. D domain and SP cluster are highlighted by *blue* and *red lines*, respectively. The putative nuclear localization signals (NLS) were predicted and pointed out by the* orange triangles* at the* bottom*. **b** Phylogenetic relationships of PtrWRKY73 proteins from *P. trichocarpa* and selected species. Sequences were from *N. tabacum* (NtWRKY1), *N. attenuate* (NaWRKY3), *Hordeum vulgare* (HvWRKY19, HvWRKY28), *V. vinifera* (VvWRKY1), *P. tremula* x *P. Alba* (PtWRKY23), *Medicago truncatula* (MtSTP), *Oryza sativa* (OsWRKY1), *Triticum aestivum* (TaWRKY1), *Capsicum annuum* (CaWRKY1), *P. trichocarpa* (PtrWRKY89), *A.* *thaliana* (AtWRKY3, AtWRKY4, ATWRKY25, AtWRKY28, ATWRKY33, AtWRKY46, AtWRKY50, AtWRKY51, AtWRKY53, ATWRKY54, ATWRKY70). And the accession numbers were described in Supplementary Table 1 (color figure online)

A cDNA fragment encoding *PtrWRKY73* was isolated from *P. trichocarpa* using gene-specific primers designed based on the sequences of the *P. trichocarpa* genome database. The length of the nucleotide sequence of *PtrWRKY73* was 1,776 bp, encoding a protein of 591 amino acid residues with a predicted theoretical isoelectric point and protein molecular weight of 7.2 and 64.93 kD. Sequence analysis showed that the deduced amino acid sequence of PtrWRKY73 contains two WRKY domains in N-terminus and C-terminus followed by a C-terminal Cys2/His2-type zinc-finger motif (Fig. 2a) and belonged to group I WRKY members. Previous studies have demonstrated that the D domain, which includes a cluster of basic residues in the upstream of the LxL motif ([K/R]1-2-x2-6-[L/I]-x-[L/I]), is a MAPK-docking site considered to define the selectivity by interacting with MAPKs (Reményi et al. 2006; Ishihama and Yoshioka 2012), and clustered serine–proline residue (SP cluster) is a minimal consensus motif for MAPK phosphorylation (Sharrocks et al. 2000). The D domain and SP cluster are conserved in the N-terminal regions of several group I WRKYs, inferring a functional importance (Sharrocks et al. 2000; Reményi et al. 2006). A deduced D domain was found in amino acid positions 78–87 of PtrWRKY73 protein and an SP cluster was adjacent to the N-terminal side of the D domain (Fig. 2a). Compared with other WRKY proteins, PtrWRKY73 had high identity with group I WRKY proteins from hybrid poplar [(*P. tomentosa* × *P. bolleana*) × *P. tomentosa*] (PtoWRKY1, 97.65 %), *Theobroma cacao* (TcWRKY33, 69.47 %), *Jatropha curcas* (JcWRKY07, 68.70 %) and *Vitis vinifera* (VvWRKY33, 65.27 %), but shared low sequence identity with AtWRKY33 (38.40 %) (Fig. 2a).

The phylogenetic tree based on the classification method of Eulgem et al. (2000) showed that PtrWRKY73 belongs to group I of the WRKY superfamily (Fig. 2b). PtrWRKY73 was allocated to group I with the closest members being NtWRKY1 from tobacco (*Nicotiana tabacum*) and AtWRKY33 from *Arabidopsis*, which showed involvement of plant defense responses (Yamamoto et al. 2004; Zheng et al. 2006). These results suggested that PtrWRKY73 might also play a role in resistance to pathogens in poplar.

### Tissue-specific expression patterns of *PtrWRKY73* in poplar

**Fig. 3:**
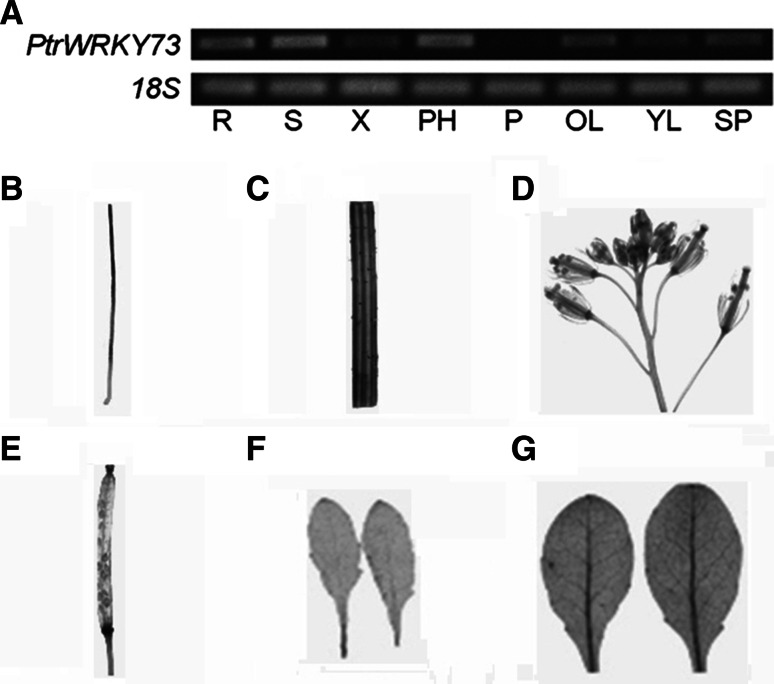
Expression profiles of PtrWRKY73 in *P. trichocarpa*. **a** Semi-quantitative PCR examined-*PtrWRKY73* transcript levels in different tissues. Poplar *18S* expression was used as a control. Total RNA was isolated from roots (R), stems (S), xylem (X), phloem (PH), petioles (P), old leaves (OL), young leaves (YL) and sprouts (SP). *PtrWRKY73* promoter driving *GUS* expression in various tissues of *A. thaliana* including roots (**b**), stems (**c**), inflorescence (**d**), siliques (**e**), young leaves (**f**) and old leaves (**g**) of 4-week-old plants

*PtrWRKY73* expression in different tissues of *P. trichocarpa* was analyzed by semi-quantitative reverse transcription (RT)-PCR. Accumulation of *PtrWRKY73* mRNA was detected in all tissues examined except in petiole (Fig. 3a). The expression levels in roots, stems and phloem were higher than in xylem, sprouts and leaves. Interestingly, *PtrWRKY73* preferentially accumulated in old leaves (the leaves from the 5–6th node) rather than in young leaves (the leaves from the 2–3rd node).

To further investigate the spatial and temporal expression patterns of *PtrWRKY73*, a 1,447 bp *PtrWRKY73* promoter fragment was isolated from the genome of *P. trichocarpa* to fuse with *GUS* reporter gene of the pCXGUS-P vector (Chen et al. 2009) to generate the *pPtrWRKY73:GUS* construct, which was introduced into wild-type *A. thaliana*. Histochemical GUS staining showed that GUS activity was detected in roots, stems, flowers, siliques and leaves of transformed plants (Fig. 3b–g). As shown in Fig. 3b, GUS expression was detected in roots except the tips. GUS activity was also found in various floral organs such as the calyx, stigmatic papillae and abscission zone (Fig. 3d). At the late stages of silique development, GUS activity staining was slightly maintained in stigmatic papillae and abscission zone (Fig. 3e). In addition, during the leaf aging process, *PtrWRKY73* transcript levels also gradually increased up to the point of leaf senescence (Fig. 3f, g).

### PtrWRKY73 is localized in the nucleus and functions as a potential transcriptional activator

**Fig. 4:**
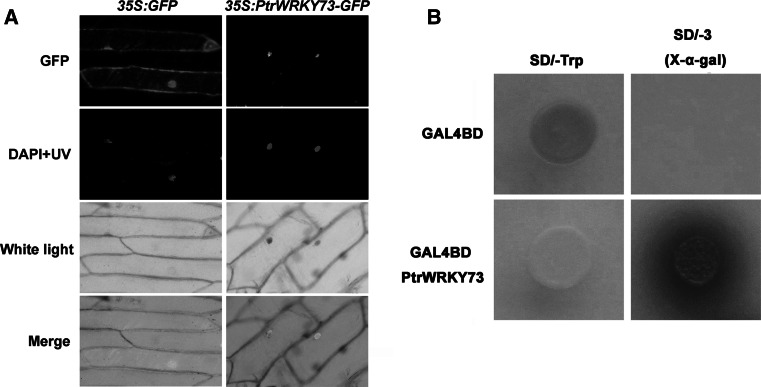
Nuclear localization and transcriptional activity of PtrWRKY73. **a** *35S*::*GFP* was served as control. *Bars* 100 mm. **b** Transcriptional activation analysis of PtrWRKY73 fused with the GAL4 DNA-binding domain (GAL4BD) showed that PtrWRKY73 had the ability to activate the expression of the Trp and α-Gal reporter genes in yeast

Sequence analysis using PSORT II Prediction (http://psort.hgc.jp/form2.html) showed that the predicted PtrWRKY73 contains two putative nuclear localization signals (299PTKKKVE306 and 392PEAKRWK398) (Fig. 2a). To verify its subcellular localization, PtrWRKY73 protein was fused to green fluorescent protein (GFP) under the control of the constitutive CaMV 35S promoter. The resulting vector was transformed into onion epidermal cells by *Agrobacterium*-mediated transformation and the result showed that the fusion protein was localized in the nucleus (Fig. 4a). In contrast, the 35S:GFP protein occurred throughout the cells including cytoplasm and the nucleus (Fig. 4a). These results indicated that PtrWRKY73 has a nuclear localization.

To further examine whether PtrWRKY73 has transcriptional activity in vivo, *PtrWRKY73* ORF was fused with the GAL4 DNA-binding domain in the pGBKT7 and transformed into Y2HGold yeast (*Saccharomyces cerevisiae*) cells. All the transformants containing pGBKT7-PtrWRKY73 grew well on selective medium without tryptophan (SD/-Trp) and SD/-Trp/-Ade/-His mediums, and these yeast cells were stained blue in X-gal solution. In contrast, the yeast cells with the negative control pGBKT7 only grew on SD/-Trp medium (Fig. 4b). These results demonstrated that PtrWRKY73 functions as a transcriptional activator.

### Constitutive expression of *PtrWRKY73* in *Arabidopsis* resulted in enhanced resistance to *Pst*DC3000 but more susceptibility to *Botrytis*

**Fig. 5:**
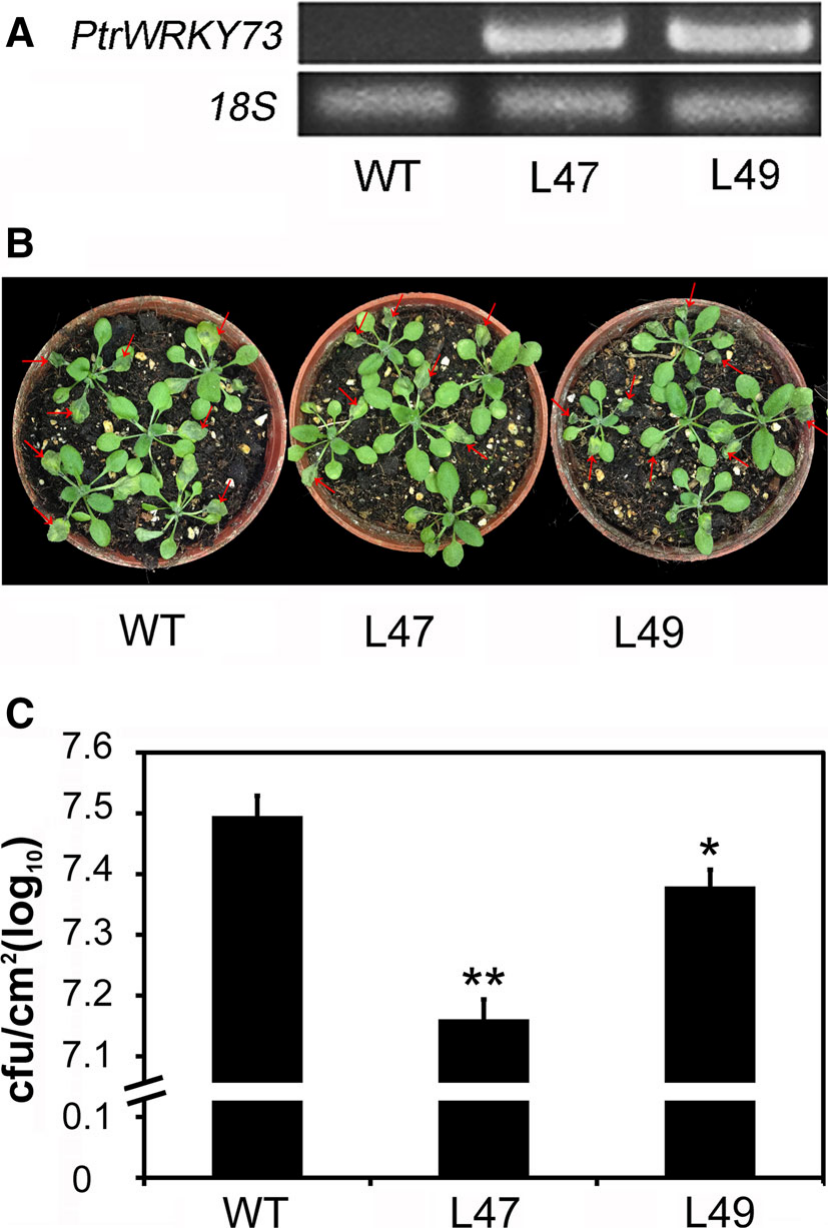
*PtrWRKY73* overexpressing *Arabidopsis* plants showing resistance to *Pst*DC3000. **a** *PtrWRKY73* expression in transgenic and wild-type plants. **b** Phenotypes of wild-type and transgenic plants after 3 days of *Pst*DC3000 infection.* Red arrows* indicate the inoculated leaves. **c** Growth of *Pst*DC3000 in *planta* 3 days after inoculation. Values are means of three replicates. *Error bars* indicate standard deviation.* Asterisks* indicate a statistically significant difference between control and transgenic plants (*P* < 0.05 by Student’s *t* test) (color figure online)

To examine the role of PtrWRKY73 in biotic plant defense, transgenic *Arabidopsis* plants overexpressing *PtrWRKY73* were examined. Two independent lines (L47 and L49) with high *PtrWRKY73* expression were selected and their homozygous progenies containing a single insert were used for further experiments (Fig. 5a). To elucidate the molecular basis of PtrWRKY73 in disease resistance in *Arabidopsis*, we examined resistance of the transgenic plants to *P. syringae* pv tomato strain DC3000 (*Pst*DC3000). Three days after infection, transgenic lines exhibited weakly enhanced resistance to *Pst*DC3000 as compared to wild-type plants (Fig. 5b). However, growth of *Pst*DC3000 was significantly lower in leaves of transgenic lines than that of the wild-type control (Fig. 5c).

**Fig. 6:**
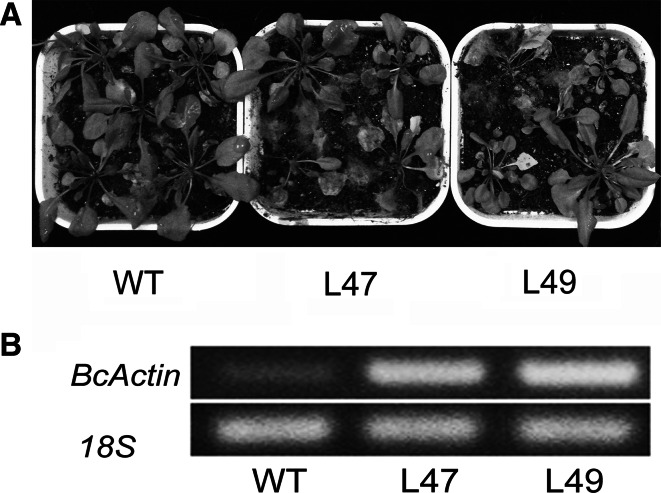
*PtrWRKY73* overexpressing *Arabidopsis* plants showing susceptibility to *Botrytis*. **a** Disease response of inoculated plants at 10 days after *Botrytis* treatment. **b** Accumulation of the *Botrytis* *Actin* mRNA in inoculated plants. *18S* was included as a control

We also analyzed the responses of transgenic plants overexpressing *PtrWRKY73* to the necrotrophic fungal pathogen *B. cinerea*. Disease symptoms in plants were quantified 10 days after inoculation. Transgenic lines tested displayed severe necrosis or death of the leaf tissue and the hyphae of *B. cinerea* flourished on the decayed tissues, whereas only several dead leaves were found in wild-type plants (Fig. 6a). Because the *Botrytis* *Actin* gene was previously shown to be constitutively expressed during plant infection (Benito et al. 1998), transcript levels were used indicative of the rate of fungal growth *in planta*. Fungal biomass in leaves was also quantified for wild-type and transgenic *35S:PtrWRKY73* plants (Fig. 6b). Semi-quantitative RT-PCR demonstrated that significantly increased levels of fungal DNA were detected from transgenic *35S:PtrWRKY73* plants compared with the wild-type control. These results indicated that overexpression of *PtrWRKY73* enhanced sensitivity to *Botrytis* in *Arabidopsis*.

### PtrWRKY73 regulating SA-related genes affecting plant defense

**Fig. 7:**
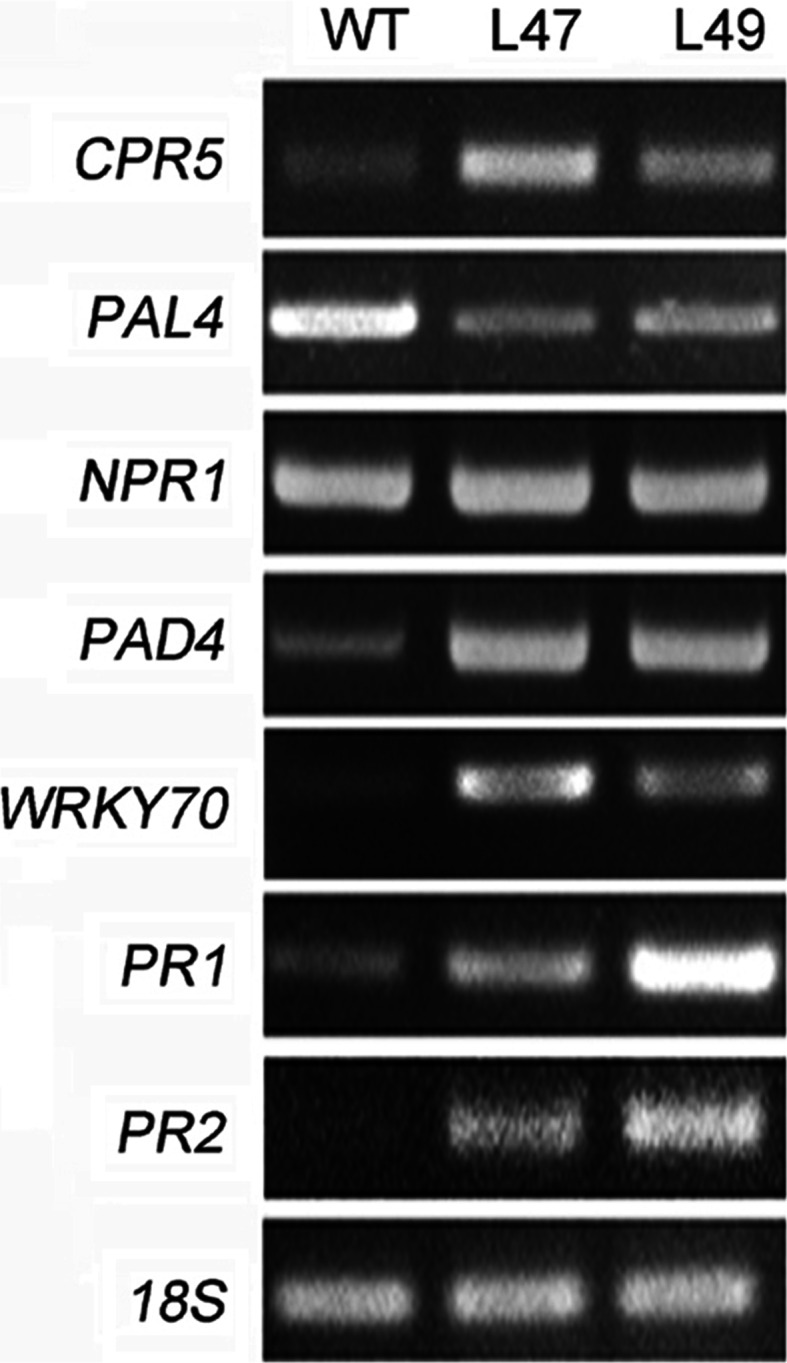
Expression of genes involved in SA signaling pathways in *Arabidopsis* overexpressing *PtrWRKY73* as compared to wild-type plants. *18S* expression was used as a control

*PtrWRKY73* was induced by exogenous SA and overexpression of *PtrWRKY73* enhanced resistance to *Pst*DC3000, but susceptibility to *Botrytis*, indicating that PtrWRKY73 is involved in SA-mediated defense pathway. Semi-quantitative RT-PCR was used to determine the expression levels of pathogenesis-related genes in *PtrWRKY73* overexpressor lines. Results show that *PR1* and *PR2* mRNAs accumulated to directly enhance resistance to *Pst*DC3000 (Fig. 7). Although *NPR1* acts as a modulator of *PR* gene expression, overexpression of *PtrWRKY73* did not affect the accumulation of *NPR1* transcripts. *WRKY70* which acts as an activator of *PR*s (Li et al. 2004) had higher expression levels in L47 and L49 compared with wild-type plants. *PAD4*, required for SA signaling amplification (Jirage et al. 1999), had increased transcript levels in transgenic plants. In addition, *CPR5* whose mutation showed constitutive expression of systemic acquired resistance (SAR) (Bowling et al. 1997) also exhibited a higher level of mRNA in transgenic *35S:PtrWRKY73* lines than the wild-type control (Fig. 7). Interestingly, the expression of SA biosynthesis-related gene *PAL4* was reduced in the transgenic lines (Fig. 7). These results indicate that overexpression of *PtrWRKY73* can impact transgenic plant defense through regulating of SA-related gene expression.

## Discussion

Recently, poplar genome sequence that has been completed (Tuskan et al. 2006) includes 100 putative WRKY genes (Jiang et al. 2014). Some WRKY genes have been cloned and characterized, for example, misexpression of *PtWRKY23* from poplar (*P. tremula* × *P. alba*) resulted in more susceptibility to *Melampsora* infection in transgenic plants (Levée et al. 2009). An SA-induced *PtoWRKY60* enhanced resistance to the fungus, *Dothiorella gregaria*, in transgenic Chinese white poplars (Ye et al. 2014). Our previous work showed *PtrWRKY73*, a pathogen-inducible gene (Jiang et al. 2014), encoded a typical WRKY protein with two WRKY domains (Fig. 2a) belonging to group I (Eulgem et al. 2000). In agreement with conserved clustered proline-directed serines (SP cluster) in group I WRKYs (Ishihama and Yoshioka 2012), the existence of the D domain and SP cluster on the PtrWRKY73 might provide an MAPK-docking site and phosphorylation targets of mitogen-activated protein kinase (MAPK) (Reményi et al. 2006;Ishihama and Yoshioka 2012), suggesting that PtrWRKY73 activity could be regulated by phosphorylation (Fig. 2a). In addition, two predicted nuclear localization signals (NLS) (Fig. 2a) strongly suggests that PtrWRKY73 protein is translocated into the nucleus to regulate gene expression. The following subcellular localization assay also supports this hypothesis (Fig. 4a).

Although the phylogenetic tree indicates that the closest homologue of PtrWRKY73 in *Arabidopsis* is AtWRKY33 (Fig. 2b) with both being inducible by SA (Zheng et al. 2006), the identity between these two proteins was only 38.40 % (Fig. 2a) and the most similar region mainly distributed at the C-terminal (Data not shown). Therefore, we assume that PtrWRKY73 and AtWRKY33 might play different roles in plant pathogen resistance. It has been demonstrated that *AtWRKY33* was required for resistance to necrotrophic fungal pathogens (Zheng et al. 2006). Whereas our findings showed that overexpression of *PtrWRKY73* could enhance the resistance to the biotrophic bacterial leaf pathogen *Pst*DC3000 (Fig. 5), but be impaired in defense responses to the necrotrophic fungal pathogen *Botrytis* (Fig. 6). These results suggested that PtrWRKY73 might be involved in SA-mediated plant defense.

According to their lifestyles, plant pathogens are generally divided into necrotrophs and biotrophs. In addition, many plant pathogens exhibit both lifestyles depending on the stage of their life cycle and are referred to as hemibiotrophs (Pieterse et al. 2009). SA-mediated signaling pathways have been demonstrated to play critical roles in resistance to biotrophic and hemibiotrophic pathogens, such as *P. syringae* pv tomato strain DC3000 (*Pst*DC3000). When plants are invaded by biotrophs and hemibiotrophs, some lipase-like genes such as *PAD4* participate in a positive regulatory loop that increases SA levels (Jirage et al. 1999). Pathogen-induced SA accumulation changed the cellular redox potential by rapidly increasing levels of ROS, resulting in translocation of NPR1 to activate pathogen resistance genes, such as *PR*s (Uknes et al. 1992; Chen et al. 1993; Baker and Orlandi 1995; Lamb and Dixon 1997; Klessig et al. 2000; Dong 2004; Loake and Grant 2007). In *Arabidopsis*, AtWRKY70, an activator downstream of *NPR1*, positively regulated the SA signaling pathway associated with induction of *PR1*, *PR2* and *PR5* to enhance resistance to *Pst*DC3000 (Li et al. 2004). In this study, *PtrWRKY73* overexpressors had high *PAD4* transcript levels compared with wild-type plants (Fig. 7), indicating that PtrWRKY73 positively regulates the SA amplification loop. Constitutive expression of *PtrWRKY73* did not lead to increased *NPR1* transcript levels but enhanced accumulation of *WRKY70* mRNA (Fig. 7), contributing to *PR1* and *PR2* accumulation (Fig. 7). Taken together, these results suggest that PtrWRKY73 positively modulates defense signal transduction pathways independent of NPR1.

Overexpression of defense-related genes probably requires high energy levels and nutrients, resulting in morphological changes in plants. In *Arabidopsis*, overexpression of *WRKY70* resulted in plants smaller in size than the controls, and transgenic plants also exhibited changes in morphology with lancet shaped and slightly twisted leaves (Li et al. 2004). In transgenic *35S:PtrWRKY73* plants, SA-related genes such as *PR*s were significantly induced but no change in morphology was found in transgenic plants compared with wild-type plants (data not shown). At the molecular level, the phenylalanine ammonia-lyase gene (*PAL4*), involved in SA biosynthesis, had reduced significantly (Fig. 7). In addition, *CPR5*, whose mutation constitutively expressed systemic acquired resistance (SAR) (Bowling et al. 1997), was activated in transgenic *35S:PtrWRKY73* plants (Fig. 7). These results indicate that a feedback mechanism might exist, that inhibits excessive SA signaling by accumulation of *CPR5* mRNA and at the same time, a reduction in *PAL4* expression level impairs the negative effect of energy and nutrition consumption in transgenic plants.

### **Author contributionstatement**

YJ and KL designed the research. YD, YJ, SY, ZL, YH and SY performed the experiments. AK, YJ and KL wrote the paper. All authors discussed the results and approved the final manuscript.

## Electronic supplementary material

Below is the link to the electronic supplementary material.

Supplementary material 1 (DOC 52 kb)
