# Supplementary material for: PtrWRKY73, a salicylic acid-inducible poplar WRKY transcription factor, is involved in disease resistance in Arabidopsis thaliana
Source: Plant Cell Rep. 2015 Jan 28;34(5):831–41. doi: 10.1007/s00299-015-1745-5 (PMC4405351; doi:10.1007/s00299-015-1745-5)
Supplement: Supplementary file 1 — Supplementary material 1 (DOC 52 kb) [file 299_2015_1745_MOESM1_ESM.doc]

**PtrWRKY73, a salicylic acid-inducible poplar WRKY transcription factor, is involved in disease resistance in *Arabidopsis thaliana***

**Authors:**
Yanjiao Duan^1#^, Yuanzhong Jiang^1#^, Shenglong Ye^1^, Abdul Karim^1^, Zhengyi Ling^1^, Yunqiu He^1^, Siqi Yang^1^, Keming Luo^*1,2^ --------------------------------------------------------------------------------------------------------^1-^Key Laboratory of Eco-environments of Three Gorges Reservoir Region, Ministry of Education, Chongqing Key Laboratory of Transgenic Plant and Safety Control, Institute of Resources Botany, School of Life Sciences, Southwest University, Chongqing 400715, China

^2-^Key Laboratory of Adaptation and Evolution of Plateau Biota, Northwest Institute of Plateau Biology, Chinese Academy of Sciences, 810008 Xining, China

* Corresponding author ([kemingl@swu.edu.cn](mailto:kemingl@swu.edu.cn)).

^#^ These authors contributed equally to this work.

Address: No. 1, Tiansheng Road, Beibei, Chongqing 400715, China.

| **Supplementary Table 1. The common names and accession numbers of the WRKY genes** | |
| --- | --- |
| Common name | Accession number |
| PtoWRKY1 | AEV66272 |
| TcWRKY33 | XP_007017012 |
| JcWRKY07 | AGJ52153 |
| VvWRKY33 | XP_002272040 |
| NtWRKY1 | BAA82107 |
| NaWRKY3 | AAS13439 |
| HvWRKY19 | DQ840418 |
| HvWRKY28 | DQ863112 |
| VvWRKY1 | AY585679 |
| MtSTP | HM622066 |
| OsWRKY1 | AAF23898 |
| TaWRKY1 | EU665424 |
| CaWRKY1 | ABP24358 |
| PtrWRKY89 | Potri.006G109100 |
| ATWRKY33 | AT2G38470 |
| ATWRKY25 | AT2G30250 |
| ATWRKY70 | AT3G56400 |
| ATWRKY54 | AT2G40750 |
| AtWRKY4 | AT1G13960 |
| AtWRKY3 | AT2G03340 |
| AtWRKY28 | AT4G18170 |
| AtWRKY50 | AT5G26170 |
| AtWRKY51 | AT5G64810 |
| AtWRKY46 | AT2G46400 |
| AtWRKY53 | AT4G23810 |

| **Supplementary Table 2. Primers for semi-qRT-PCR in this study.** | |
| --- | --- |
| AtPAL4-RT-F | 5'>ATCAGCAGTGAGTCAGGTGG<3' |
| AtPAL4-RT-R | 5'>CTTGAGACATTCCAACAACG<3' |
| AtNPR1-RT-F | 5'>TGACTAGCCTCGAGCCTGAC<3' |
| AtNPR1-RT-R | 5'>GCAAGAGTCTCACCGACGAC<3' |
| AtPR1-RT-F | 5'>TCGTCTTTGTAGCTCTTGTAGGTG<3' |
| AtPR1-RT-R | 5'>TTCATTAGTATGGCTTCTCGTTCA<3' |
| AtPR2-RT-F | 5'>CCACCAATGTTGATGATTCTTCT<3' |
| AtPR2-RT-R | 5'>ATACTCATCCCTGAACCTTCCTT<3' |
| AtPAD4-RT-F | 5'>CTATTCTGAGGTCAGCTGAG<3' |
| AtPAD4-RT-R | 5'>CGCATAACTCTCGAATGGAAC<3' |
| 18S-F | 5'>CGAAGACGATCAGATACCGTCCTA<3' |
| 18S-R | 5'>TTTCTCATAAGGTGCTGGCGGAGT<3' |
| PtrWRKY73-RT-F | 5'>AGGGCACCACCTACAGCA<3' |
| PtrWRKY73-RT-R | 5'>AATGGGTAATGGATTTGACG<3' |
| AtCPR5-RT-F | 5'>GAGAATCGAGGATGCTACC<3' |
| AtCPR5-RT-R | 5'>AGGTGATACATAGCGAAGTC<3' |
| AtWRKY70-RT-F | 5'>CATACATAGGAAACCACACG<3' |
| AtWRKY70-RT-R | 5'>CTCCAAACACCATGAGATCC<3' |
